# Supplementary material for: Detection of malaria parasites in dried human blood spots using mid-infrared spectroscopy and logistic regression analysis
Source: Malar J. 2019 Oct 7;18:341. doi: 10.1186/s12936-019-2982-9 (PMC6781347; doi:10.1186/s12936-019-2982-9)
Supplement: Supplementary file 1 — Additional file 1. Detection of five Plasmodium species from dried blood spots (DBS) by nested PCR method. [file 12936_2019_2982_MOESM1_ESM.docx]

**Additional material**

**Detection of five *Plasmodium* species from Dried Blood Spots (DBS) by nested PCR method.**

**DNA extraction**: Three spots of individual sample from DBS were cut into small pieces with scissors and transferred into 2.0 ml of micro-centrifuge tube, 200µl of Tris-EDTA (TE) buffer was added in the tubes and incubated at 37ºC overnight. The DNA was extracted from blood using Quick-DNA Miniprep plus ZYMO research kit as per manufacturer’s instructions.

**Detection of *Plasmodium* species**: The presence of *Plasmodium* species i.e *Plasmodium falciparum, Plasmodium ovale, Plasmodium vivax, Plasmodium malariae* and *Plasmodium knowlesi* was detected using nested PCR method targeting 18S ssRNA genes according to Sounou *et al* following slightly modification. For the first PCR reaction 10µl of DNA was used, in which the fragments was amplified using rPLU1 and rPLU5 primers which target between 1600 – 1700bp. For the second PCR about two microliters of the first PCR product was used as a template for the second PCR reaction in which the species specific primer pairs (rFAL1-rFAL2, rOVA1-rPLU2, rVIV1-rVIV2, rMAL1-rMAL2, and Pmk8-Pmkr9) were used for each of the five separate reactions. The amplification for all reactions were carried on 20µl of PCR master mix consisting of 0.5µM of each primer, 2X buffer, 3mM MgCl_2_, 0.5mM of each of the four dioxyribonucleotide triphosphate (dNTP) and 0.3 units of Taq DNA polymerase.

The DNA amplification was carried out under the following conditions, initial denaturation temperature of 94ºC for 2 min, followed by 35 amplification cycles at 94ºC for 30 sec, 62ºC for 30 sec, and 72ºC for 1 min, followed by final extension at 72ºC for 7 min. The concentration of the second PCR primers and other constituents were identical to those used for the first PCR. The second PCR amplification conditions were identical to those of the first PCR except that the amplification for the second PCR was performed over 40 cycles. The PCR products were analyzed after electrophoresis in 2% agarose gel stained with ethidium bromide. DNA bands were visualized under ultraviolet light using Kodack Logic 100 imaging system.

**Table S1**: *Plasmodium species* amplified fragments sizes.

| **Species** | **Fragment size (bp)** |
| --- | --- |
| *Plasmodium falciparum* | 206 |
| *Plasmodium vivax* | 121 |
| *Plasmodium ovale* | 226 |
| *Plasmodium malariae* | 145 |
| *Plasmodium knowlesi* | 153 |

**References**

1. Zhou X, Huang J-L, Njuabe MT, Li S-G, Chen J-H, Zhou X-N. A molecular survey of febrile cases in malaria-endemic areas along China-Myanmar border in Yunnan province, People’s Republic of China. Parasite. 2014;21:27.
2. Snounou G, Singh B. Nested PCR Analysis of Plasmodium Parasites. In: Doolan DL, editor. Malar Methods Protoc Methods Protoc. Totowa, NJ: Humana Press; 2002. p. 189–203.
